# Immediate self-information is prioritized over expanded self-information across temporal, social, spatial, and probability domains

**DOI:** 10.1177/17470218211004208

**Published:** 2021-04-02

**Authors:** Hyunji Kim, Arnd Florack

**Affiliations:** Department of Applied Social Psychology, Faculty of Psychology, University of Vienna, Vienna, Austria

**Keywords:** Self-representation, expanded self, information prioritization, implicit bias, individual differences

## Abstract

People construct self-representation beyond the experiential self and the self-concept can expand to interpersonal as well as intrapersonal dimensions. The cognitive ability to project oneself onto expanded selves in different time points and places plays a crucial role in planning and decision-making situations. However, no research to date has shown evidence explaining the early mechanism of how processing the experiential self-information differs from processing the expanded self-information across temporal, social, spatial, and probability domains. We report novel effects showing a systematic information prioritization toward the experiential selves (i.e., the self that is now, here, and with highest certainty) compared to the expanded selves (i.e., the self that is in the future, at a distant location, and with lower certainty; Experiments 1a, 2, and 3). Implicit prioritization biases lasted over time (Experiment 1b; i.e., 4 months) indicating a trait-like more than a state-like measure of individual differences. Different biases, however, did not consistently correlate with each other (Experiments 1a to 3) suggesting separate underlying mechanisms. We discuss potential links to the basic structure of self-representation and individual differences for implications.

Self-bias literature to date has mostly focused on comparing self- versus other-referential processing. In the last decades, researchers have shown that self-relevant information (e.g., self-owned, self-referential, or self-picked objects) is remembered better (e.g., Conway & Pleydell-Pearce, 2000; Symons & Johnson, 1997) and processed faster than other-relevant information (Keyes & Dlugokencka, 2014; Sui & Han, 2007). Recent evidence has also shown that earlier information processing biases exist toward neutral objects once associated with the self, compared to other social entities (Moradi, Sui, Hewstone, & Humphreys, 2015; Sui, He, & Humphreys, 2012; Sui, Rotshtein, & Humphreys, 2013). Self-picked or even forcefully assigned-to-self objects were also preferred and evaluated more positively compared to counterpart objects (e.g., Gawronski et al., 2007). Previous evidence pointing to such consistent self-bias in cognition, however, mainly focused on comparing the self with other social entities (e.g., friend, family, colleagues, stranger or our-group members). Nevertheless, we construe ourselves not only as a social agent differentiated from other entities but also as a person living through different time points and spatial locations. Many decisions for ourselves involve transcending here and now and being able to recruit self-relevant information that stretches across different times and places. In the present study, we aimed to identify enduring characteristics of processing immediate (i.e., me here and now with a higher certainty) and expanded self-information (i.e., me far away and in the future with a lower certainty) in multiple domains (i.e., temporal, spatial, social, and probability domains) at a basic representational level.

As research on the process of self-relevant information varies in its characteristics depending on how the self-related processing is defined (Northoff, 2007, 2011, 2016), we define the self as subject for immediate self-information (experiencing self; Northoff, 2016) and the self as object for temporally, socially, spatially, and probabilistically expanded self-information (content-based self; D’Argembeau, 2013; Klein, 2012; Northoff, 2013). Thus, our studies focused on the representational structures of self as subject and object for investigating how the mechanisms differ at the basic processing level.

Understanding the function of the self-representation in multiple domains is often conceptualized within the framework of self-projection: the ability to put the immediate self into the shoes of future or past selves, another person’s mind, and counterfactual situations (Buckner & Carroll, 2007; De Brigard et al., 2013; Schacter & Addis, 2007). Accumulated evidence supports that recollecting one’s information from the past, thinking about future, and planning ahead might involve a unitary underlying mechanism (e.g., Schacter et al., 2007). However, while empirical research investigating the commonality of such a neural network is rich, examining the basic information processes and boundary conditions of various self-representations expanded to social, temporal, spatial, and probability domains is largely missing. Current literature does not give clear answers to whether different types of self-representations (i.e., immediate and expanded self-concept) show a functional similarity in information processing or whether representing self-relevant information that is expanded to different domains shows similar processing mechanisms at an earlier level. In the present study, we aimed to fill in this much-needed gap in the field.

## Immediate and expanded self-representation

At the core of our personal identity lie our self and its continuity of time and space (Ersner-Hershfield et al., 2009; Northoff, 2018). It is though, to date, unclear through what processes the stable self-concept is achieved in the midst of continuously changing surroundings. How do we maintain the stability and continuity of the self when the self is flexibly represented in a dynamic environment across time, space, and probable situations? What is the basic mechanism that successfully differentiates what is being experienced now (self as subject) and what is beyond that (self as object)? These are important questions because understanding the mental structure of the self that expands to temporal, social, spatial, or probability domain can advance the theoretical framework of self-representation into a more integrative system.

Immediately available information about the self (i.e., present feelings and attitudes) often serves as sources to infer others’ attitudes and feelings, predict behavior, and plan one’s own actions (e.g., Adolphs, 2002; Davies & Stone, 1995; Meltzoff & Brooks, 2001; Mitchell et al., 2005). Equally important is the ability to draw a sharp mental separation from the information that is not currently being experienced by the self. This cognitive skill is crucial presumably to maintain the homeostasis of the experiential self and to efficiently filter out self-irrelevant information (e.g., Burgess et al., 2003; Epley & Gilovich, 2001, 2004). In social domain, research on mentalizing with others shows that projecting oneself onto another person’s mind not only requires an “anchor” stage whereby putting oneself into another person’s shoe but also requires an “adjustment” stage whereby self-referential processing is inhibited so that what is relevant for the current self is clearly distinguished from the other-referential processing (Epley & Gilovich, 2001; Tamir & Mitchell, 2010, 2013). In principle, recruiting information from the past, future, or alternative situations undergoes a similar process as recruiting information about other people despite being intrapersonal (i.e., within a continuum of the self-identity), as opposed to interpersonal, construct in nature. Given the two-stage processes of mentalizing, it might be plausible to think that information involving the currently experienced self can be systematically selected and processed somewhat in a different fashion compared to those involving the expanded targets (i.e., other people, self in the future, self in a different location) at perhaps an early information processing stage before any further processes (e.g., evaluation, judgment) occur. In social psychology, the way our representation systematically changes as a function of subjective distance is often explained as shifts between construal levels. That is, temporally, socially, spatially, and hypothetically proximate situations are generally construed in a more concrete and contextualized manner, whereas distant situations are construed in a more abstract and general manner (Trope & Liberman, 2000, 2003, 2010). Recent evidence on spontaneous brain activity also shows that temporal, social, and spatial synchrony with the self occurs when judging an animate but not an inanimate object (Scalabrini et al., 2019), indicating that self-expansion toward other domains might be limited to animate objects. However, the relevant literature does not predict general information prioritization for a specific type of information nor suggest representational overlaps among expanded self-concepts of multiple domains.

It is thus an open question whether self-relevant information that expands to future, space, and probable situations actually shares an identical information process that differentiates the self- and other-relevant information because interpersonal comparisons occur between the self and non-self targets, whereas intrapersonal comparisons bear physical continuity of the self, thus a coexistence of the comparison targets (i.e., me now and me in the future). In social domain, the conceptual aspect of “self-bias” makes it clear that self-relevant information is prioritized over other-relevant information but will there be a systematic bias for processing information for temporal, spatial, and probable selves? Based on previous evidence, thinking about another person’s mind, one’s own future and recollecting past events equally involve an episodic simulation process (Schacter et al., 2007). Yet, through what process specific information is being selected for further simulation processes and whether each selection criterion differs across multiple domains are not clear.

One possible selection criterion is the level of closeness to the experiential self. When a given set of information greatly concerns the self that is here and now, it might be prioritized over other types of self-relevant information that is more stretched in time and space because the immediate self-information facilitates the episodic simulation process. Accumulated evidence has shown that the extent to which people feel how connected they are with future selves or with other people often strongly correlates with increased quality of the episodic simulation (e.g., Ames, 2004; Bartel & Rips, 2010; Mitchell et al., 2006; Trope & Liberman, 2000). That is, the more your experiential self can be identified with your future self or with another person, the more vivid and fluent your simulation becomes. Fluent mental simulations enable us to plan our actions more efficiently and make choices closer to serving our current needs (e.g., Faude-Koivisto et al., 2008; Taylor & Pham, 1996). Thus, all self-relevant information might not be equally important but rather, systematically prioritized according to closeness to the experiential self for maintaining and pursuing current goals.

Taken together, we propose an overarching system that differentiates immediate and expanded self-information across social, temporal, spatial, and probability domains by prioritizing information that is closer to the experiential self. In the following section, we further elaborate our prioritization claim for immediate over expanded self-information based on the salience account.

## Immediate self-information may be more salient than expanded self-information

We propose that self-relevant information that reflects the experiential self (i.e., me here and now) might receive a specific information-processing advantage over self-relevant information that reflects the self that goes beyond the experiential self (i.e., me far away and in the future), due to higher salience. Below, we elaborate our rationale based on the identity-value model and introduce supporting evidence from the recent research on attention modulation for self-relevant objects.

One grounded reason for the general self-bias in information processing is that self-relevant information is most valuable (e.g., Berkman, Hutcherson, et al., 2017; Northoff & Hayes, 2011). The identity-value model specifically conceptualizes this claim as a system and predicts that a mental representation overlapping with one’s current self-identity will be salient because that information holds greater subjective value to the self (for a review, see Berkman, Livingston, & Kahn, 2017). This view stems from the assumption that the self is constructed by the collection of subjective values. As such, the structure of one’s own self-representation reflects one’s current goals and motivation. The self-identity also constantly evolves according to the way we form and evaluate values and goals in various decision contexts. Applying this model to processing self-relevant information, we argue that because immediately experienced self-, compared to expanded self-, information would be more likely to echo one’s current self-identity as it is useful for satisfying current needs and achieving proximate goals, immediate self-information becomes more salient. Although expanded self-information can be useful for planning and predicting future behavior, immediate self-information generally holds more value for providing accurate assessment of what is at stake. Indeed, the central idea of the identity-value model is that a given set of self-relevant information becomes salient once the information concerns the current self-concept. This process is highly important because such information will guide one’s behavior to achieve a goal and ultimately confer benchmarks for regulating the self. We argue that the individual tendency for information prioritization arises from the accumulated experiences of lifetime whereby a repeated “on-line” adjustment toward salient self-relevant information occurs. Consequently, information relevant to the immediate self, compared to the expanded self, would serve to regulate one’s behavior and achieve goals via salience, signaling for information prioritization. Hence, we hypothesized that immediate self-information would be prioritized over expanded self-information across temporal, spatial, and probability domains that offer dynamic decision contexts where one’s self-identity can be constructed in.

Some evidence supporting that self-relevant information is perceived more salient than other-relevant information comes from a self-bias research with a focus on attention modulation. Recent findings on self-bias using a simple object-label matching task have shown that self- (vs. other-) relevant information attracts and modulates attention to accelerate information processing and integration (Humphreys & Sui, 2016; Sui & Humphreys, 2017). This perceptual self-bias is not caused by the difference in word length, concreteness, or familiarity of a given set of information (Sui et al., 2012), nor by the memory order effect (Kim et al., 2019), and learning or working memory effects (Kolvoort et al., 2020). Further research has shown that neutral shapes (i.e., circle, triangle, diamond) can gain perceptual salience comparable to shapes that are physically made perceptually noticeable (e.g., colored shapes), once associated with the label “self” (vs. “other”). Both self-associated and colored shapes modulated attention by recruiting the same neural networks, facilitating perceptual prioritization over other associated and gray scale shapes (Liu & Sui, 2016; Sui et al., 2013). Self-associated information also showed an enduring processing advantage of temporal processing, evident in the temporal measures of resting-state electroencephalography (EEG; Kolvoort et al., 2020; Wolff et al., 2019).

Building on previous findings, we argue that the strength of the information prioritization will increase as a function of closeness to the experiential self because the more representational overlap you have between the experiential self and the target information, the more perceptual salience the target information would gain, leading to stronger prioritization. In other words, self-relevant information that shares the largest representational overlap with the currently experienced self (i.e., immediate self-information) will be prioritized over self-relevant information that shares a smaller representational overlap (i.e., expanded self-information).

Besides our prediction on the general prioritization effect, we also predict that the extent to which people prioritize immediate over expanded self-information would be consistent over time because the prioritization effects rise as individual implicit prioritization biases that are accumulated through experiences of life time. However, whether the magnitudes of the prioritization effect in multiple domains would correlate with each other is an open question. People in fact show large individual differences for constructing the self-identity depending on whether they identify themselves with other possible selves (e.g., ideal-self, ought-self; Self-Discrepancy Theory; Higgins, 1987). Likewise, we believe that the extent to which people identify themselves with expanded selves (e.g., the self that is far away, or in the future) would differ person to person, leading to distinct individual differences in prioritizing immediate over expanded self-information for each domain. If consistent cross-domain correlations were found, the prioritization effects might reflect a general cognitive ability rather than a specific processing advantage for self-relevant information. Therefore, we also tested inter-temporal and inter-domain consistencies of the prioritization effects to answer these questions.

In the present research, we tested the prioritization hypothesis across temporal, social, spatial, and probability domains, given that our self-representation does not only expand to another social entity but also to the self into different time points, locations, or probable situations. We expected that a systematic prioritization effect would occur for immediate over expanded self-information as a function of closeness to (i.e., overlap with) the experiential self. We also hypothesized a consistent inter-temporal but inconsistent inter-domain trends for the prioritization effects at the individual level.

## Present research

To demonstrate the prioritization effect, we utilized a recently developed paradigm: The shape–label matching task. This paradigm can test whether a neutral stimulus (e.g., arbitrary geometric shapes; Sui et al., 2012) is given information prioritization once it is associated with specific social information (e.g., immediate self- and socially, temporally, spatially, and probably expanded self-labels) within a set of given information. This paradigm is effective for ruling out common confounds such as familiarity or repeated exposure for self- and other-relevant stimuli (i.e., faces, names) by testing the effect of associations made to arbitrary shapes for which participants have no such biases. Despite diverging evidence on which level of cognitive processes such self-bias occurs (e.g., Falbén et al., 2019; Humphreys & Sui, 2015; Stein et al., 2016), the prioritization effect using the shape–label matching paradigm has been shown to be highly robust across individuals and culture (e.g., Jiang et al., 2019; Woźniak & Knoblich, 2019; Stolte, Humphreys, Yankouskaya, & Sui, 2017; for reviews, see Sui & Gu, 2017; Sui & Humphreys, 2017; Sui & Rotshtein, 2019).

We predicted that arbitrary geometric shapes that convey the conceptual representation of immediate self-information (e.g., shapes associated with the labels such as “myself right now,” “myself right here”) should show processing advantages (i.e., information prioritization) compared to the shapes that are associated with expanded self-information (e.g., shapes associated with the labels such as “myself in a year,” “myself far away”). We tested this hypothesis across temporal, social, spatial, and probability domains (Experiments 1a, 2, and 3) and explored whether these prioritization effects correlate with each other. We also tested whether the measures for prioritization fluctuated over time (4 months; Experiment 1b). Finally, we collated our data and reported the internal consistency of the temporal perceptual self-bias (*N* = 135) for gauging individual differences and reported meta-analyses of the currently observed effects in the General Discussion.

## Experiment 1a. Prioritizing immediate social and temporal selves

The purpose of Experiment 1a was twofold. First, to test whether individual information prioritization effects last over time, we recruited a sample to test for the baseline (Time 1). Second, recently, Golubickis et al. (2017) and Kim et al. (2019) reported a novel prioritization effect toward temporally immediate over expanded self-information (e.g., myself now vs. myself in a year). Kim et al. (2019) showed that this tendency correlated with the self-bias (e.g., myself vs. stranger) at the individual level. Here, our aim was to test the same hypothesis by using a sample from a different cultural background (Austrian vs. British) and by improving an experimental design for ruling out an alternative explanation. In Kim et al. (2019), participants performed two shape–label matching tasks (i.e., social and temporal tasks) one after another as consecutive blocks per task, allowing the possibility that the observed effect might have come from a learning (i.e., training) effect rather than an information prioritization effect. That is, the effect might have been confounded by the gradual improvement of the individual task performance. In the present study, we presented two shape–label matching tasks at the same time by randomly interleaving task blocks for both social and temporal tasks to minimize the training effect.

### Method

All experiments included in the present research were ethically approved by the Institutional Review Board of the Department of Applied Psychology: Work, Education, Economy at the University of Vienna.

#### Participants

Fifty-eight participants (11 males, *M*_age_ = 20.33 years, *SD*_age_ = 2.95 years) were recruited for an exchange of course credits at the University of Vienna. Based on the effect size of the perceptual self-bias measured in Sui et al. (2012), a minimum required sample size was 26 (Jiang et al., 2019). Considering a potential dropout rate of 50% for participating in the same task at the second time point of testing (4 months later), we recruited more than twice the sample size that we needed to detect the effect with 80% power at the alpha level of .05. For our planned correlation analysis, a minimum required sample size was 46 based on the medium to high effect size (*d* = .4) drawn from Kim et al. (2019).

#### Stimuli and tasks

For each participant, six geometric shapes (hexagon, horizontal ellipse, vertical rectangle, diamond, cross, and reversed triangle, each measuring approx. 4° × 4° of visual angle) were presented above a white fixation cross at the center of the screen together with a label presented below the white fixation cross. The shapes were randomly assigned to two sets of labels: social and temporal-self labels (i.e., social labels: *Myself, Friend, Stranger*; temporal self-labels: *Right now, Tomorrow, In a year*) translated in German language (see supplementary material for all translated labels). Participants subsequently indicated whether the shape–label pairs were correct or incorrect as originally assigned. All stimuli were shown on a gray background on a 19 in. monitor (1,280 × 1,024 pixel resolution at 60 Hz). The experiment was run on a PC using *PsychoPy* (version 1.90.3).

#### Procedure

Participants were instructed to make associations between shapes and social or temporal self-labels. For instance, in the social label blocks, participants read, “Cross is myself,” “Reversed triangle is friend,” “Hexagon is stranger” and in the temporal self-label blocks, participants read “Vertical rectangle is me right now,” “Horizontal ellipse is me tomorrow,” and “Diamond is me in a year.” Participants were also verbally instructed to actively engage in imagination that the social targets described in each label were represented in the assigned shapes. The order of presenting association sentences was counterbalanced across participants. After participants had viewed the shape–label associations and thought that they knew them by heart (approximately 1–2 min), they performed two shape–label matching tasks representing two sets of labels each (i.e., social or temporal self-labels) across six blocks. Each block had either social or temporal-self labels and at the beginning of each block, participants were reminded of the shape–label associations. Each trial began with a central fixation cross presented for 500 ms, followed by a pair of a shape and a label presented for 100 ms. The label either matched or did not match the shape as assigned. After presenting a shape–label pair, the screen turned blank, allowing up to 1,200 ms for participants to respond. Participants judged whether the presented shape–label pair was a correct or incorrect pair by pressing keyboard buttons as quickly and as accurately as possible. After each trial, feedback was provided for 500 ms (*correct, incorrect, or too slow*). Participants completed six blocks (three blocks for each of temporal and social tasks; 120 trials in each block, 720 trials in total) after a practice session of 12 trials for each of the temporal and social tasks. Matching and non-matching pairs occurred equally often in random order. Participants were shown their overall accuracy and average reaction time at the end of each block.

### Results and discussion

In the shape–label matching task, we operationalized prioritization as faster processing speed without any speed and accuracy trade-off when processing learned information that binds a target shape and the correctly assigned label (Sui et al., 2012). In other words, we expected to observe advantageous processing speed in the matching trials when participants accurately identified the shape–label pair for immediate self-information. To show this, we collected “correct” responses only and performed a 2 (matching vs. nonmatching) by 3 (shape category) repeated measures analysis of variance (ANOVA) for each shape–label task to test whether the effect occurred in the matching conditions only (a significant interaction between matching condition and shape category). Next, we expected to see the effect of shape category (i.e., assigned labels to each shape) on the processing speed in the correctly identified matching trials. To show this, we performed another set of repeated measures ANOVAs with correct matching trials only. We also reported the same analyses on performance sensitivity (*d′*) to show whether the processing advantage spilled over to increased sensitivity. Finally, we calculated social and temporal self-biases by using the normalization method employed in Jiang and colleagues (2019) to compare cross-domain biases in all experiments.

#### Processing speed (RT)

Responses shorter than 200 ms were excluded. One participant was excluded due to lower than the chance level performance for an overall accuracy score. First, two separate 2 (matching vs. nonmatching) × 3 (shape category) ANOVAs on correct trials were performed (see Table 1 for the means). Significant interactions between matching condition and shape category on RTs were observed in social *F*(2, 112) = 77.49, *p* < .001, $\eta_{p}^{2}=.58$ and temporal domains, *F*(1.75, 97.91) = 32.93 (Greenhouse–Geisser corrected), *p* < .001, $\eta_{p}^{2}=.37$ , indicating that the effect of shape category depended on the matching condition. Next, following the analyses by Sui et al. (2012), we conducted one-way repeated measure ANOVAs for correct matching trials. Significant effects of shape category emerged in social *F*(2, 112) = 116.05, *p* < .001, $\eta_{p}^{2}=.68$ , and temporal domains, *F*(1.78, 99.47) = 18.12 (Greenhouse–Geisser corrected), *p* < .001, $\eta_{p}^{2}=.24$ . After controlling the false discovery rate (FDR; Benjamini & Hochberg, 1995) in multiple pairwise comparisons, significant differences were found between all social and temporal shape category comparisons (Table 1).

**Table 1.** Mean reaction times and accuracies per matching condition (matching vs. non-matching) and shape category in Experiment 1a (*N* = 57).

Task	Conditions	Shape category	Mean RT (ms)	Accuracy
Social labels	Matching	Self	634 (51)_a b_	0.90 (0.06)
		Friend	692 (55)_a c_	0.80 (0.11)
		Stranger	716 (62)_b c_	0.70 (0.12)
	Non-matching	Self	733 (61)	0.82 (0.12)
		Friend	749 (61)	0.76 (0.11)
		Stranger	738 (54)	0.79 (0.12)
Temporal self-labels	Matching	Right now	665 (46)_d e_	0.88 (0.08)
		Tomorrow	682 (49)_d f_	0.80 (0.11)
		In 1 year	706 (60)_e f_	0.76 (0.15)
	Non-matching	Right now	756 (51)	0.75 (0.12)
		Tomorrow	751 (52)	0.81 (0.10)
		In 1 year	738 (50)	0.80 (0.11)

RT = reaction time; Accuracy = proportion correct. Standard deviations appear within parentheses.

The following *p* values assigned to each subscript represent pairwise comparisons between conditions with the shared subscript. ^a,b,c,e,f^*p* < .001. ^d^*p* = .009. All *p*s were FDR-corrected.

#### Performance (*d′*)

Performance levels in the matching and mismatching conditions for each shape were combined to form a measure of performance sensitivity (*d′* = *z* [Hit rate]—*z* [False alarm rate]). Higher *d′* indicated higher sensitivity toward the target shape category. Two separate ANOVAs were conducted with three shapes associated with social or temporal-self labels as an independent measure on *d′*. Significant effects of shape category emerged for social, *F*(2, 112) = 64.75, *p* < .001, $\eta_{p}^{2}=.54$ , and temporal shape categories, *F*(2, 112) = 8.68, *p* < .001, $\eta_{p}^{2}=.13$ indicating that participants were more sensitive to immediate social and temporal-self information (*Myself: M* = 2.42, *SD* = .79 and *Right now: M* = 2.01, *SD* = .69) over expanded self-associated information (*Friend: M* = 1.72, *SD* = .81, *Stranger: M* = 1.43, *SD* = .68, *Tomorrow: M* = 1.85, *SD* = .69, and *In a year: M* = 1.69, *SD* = .78). Significant differences were found between all social and temporal shape category comparisons (FDR-corrected *p*s ≤ 0.001 for *Self*-*Friend, Friend*-*Stranger, Self*-*Stranger, Right now*-*In a year* comparisons, *p* = .026 for Tomorrow-In a year, and *p* = .049 for *Right now*-*Tomorrow* comparison). A further 2 (dimension) by 3 (shape category) ANOVA revealed a significant interaction between the dimension and shape category, *F*(2, 112) = 19.45, *p* < .001, $\eta_{p}^{2}=.26$ , indicating that people showed bigger sensitivity differences among social labels compared to temporal self-labels, implying a stronger bias for the socially immediate compared to temporally immediate shape category.

#### Social and temporal self-biases (prioritization biases)

Social and temporal self-biases were calculated by subtracting the mean RTs of the immediate self-associated shapes from the mean RTs of the expanded self-associated shapes (e.g., *Stranger*—*Self, In a year*—*Right now*) and dividing it by the sum of the two mean RTs (Jiang et al., 2019). Correlational results showed no significant correlations between social and temporal self-biases (all *r*s < .12, all *p*s > .37, see supplementary material for full correlation results) indicating that the magnitude for prioritizing immediate over expanded self-information in social domain was not associated with that of temporal domain at the individual level.

In Experiment 1a, we successfully replicated the prioritization effects in processing social and temporal self-information observed in Kim et al. (2019) in both processing speed and performance sensitivity. However, our results did not show significant correlations between social and temporal self-biases. One possible explanation is that our design minimized the training effect by randomly interleaving blocks from two tasks so that any correlational results were not confounded by the level of performance improvement. Another difference was the sample coming from different cultural backgrounds but to our knowledge, no cognitive difference for self-information processing has been reported between Austrian and British populations. Thus, our results most probably speak to the possibility that the social and temporal self-information might not be consistently processed in the same way. Given this evidence, we speculate that temporally and socially expanded self-representation might be dissociable constructs. Our result might represent the distinct intrapersonal and interpersonal nature of self-representation in temporal and social domains. However, although prioritization biases are comparable, we cannot compare the main effects of shape category between temporal and social domains because we did not use the same metrics for measuring biases. Overall, our results showed that immediate social and temporal self-information benefited from the prioritizing effect over expanded social and temporal self-information. Next, we tested the stability of the social and temporal self-biases over time of such prioritization effects at the individual level.

## Experiment 1b. Stability of prioritization effects over time

To investigate the stability of the social and temporal self-biases over time, participants who took part in Experiment 1a were invited back to take part in the identical study 4 months later.

### Method

#### Participants

Twenty-five participants (3 males, *M*_age_ = 20.24 years, *SD*_age_ = 2.16 years) returned to take part in the same experiment and received course credits as compensation at the University of Vienna. Based on our results in Experiment 1a, the power with the sample size of 25 to detect the prioritization effects observed in both social and temporal tasks was over 98% at the alpha level of.05.

#### Procedure

Participants were instructed in the same way as per Experiment 1a and the procedure remained exactly identical to Experiment 1a.

### Results and discussion

#### Processing speed (RT)

The same analyses were conducted and showed the identical results to Experiment 1a. Significant interactions between matching condition and shape category on RTs were observed in the social *F*(2, 48) = 23.87, *p* < .001, $\eta_{p}^{2}=.50$ and temporal domains, *F*(2, 48) = 14.96, *p* < .001, $\eta_{p}^{2}=.38$ , indicating that the effect of shape category depended on the matching condition. Two one-way repeated measure ANOVAs for correct matching trials revealed significant effects of shape category on RTs in the social *F*(2, 48) = 43.47, *p* < .001, $\eta_{p}^{2}=.64$ , and temporal domains, *F*(2, 48) = 8.62, *p* = .001, $\eta_{p}^{2}=.26$ (see Table 2 for means and multiple comparisons).

**Table 2.** Mean reaction times and accuracies as a function of matching condition (matching vs. non-matching) and shape category at T2 in Experiment 1b (*N* = 25).

Task	Conditions	Shape category	Mean RT (ms)	Accuracy
Social labels	Matching	Self	611 (45)_a b_	0.90 (0.11)
		Friend	682 (51)_a c_	0.81 (0.12)
		Stranger	684 (56)_b c_	0.73 (0.15)
	Non-matching	Self	700 (62)	0.85 (0.09)
		Friend	711 (64)	0.83 (0.09)
		Stranger	708 (57)	0.83 (0.09)
Temporal self-labels	Matching	Right now	638 (50)_d e_	0.85 (0.10)
		Tomorrow	645 (44)_d f_	0.88 (0.19)
		In 1 year	671 (48)_e f_	0.79 (0.12)
	Non-matching	Right now	700 (57)	0.83 (0.08)
		Tomorrow	699 (61)	0.86 (0.09)
		In 1 year	690 (60)	0.85 (0.10)

RT = reaction time; Accuracy = proportion correct. Standard deviations appear within parentheses.

The following *p* values assigned to each subscript represent pairwise comparisons between conditions with the shared subscript. ^a^,^b^*p* < .001, ^c^*p* = .730. ^d^*p* = .297 ^e^*p* = .006 ^f^*p* = .008. All *p*s were FDR-corrected.

#### Performance (*d′*)

Participants’ *d′* was calculated for both times. Overall mean *d′*s indicated that participants’ task sensitivity improved over time (see supplementary material for means and comparisons). Performances at the individual level were largely correlated with each other across all shape categories between T1 and T2. Interestingly, the performance sensitivity scores for temporal shape categories were highly consistent over time, *Right now, r*(23) = .72, *p* < .001; *Tomorrow, r*(23) = .45, *p* = .024, *r*(23) = .40, *p* = .049, whereas social shape categories were only partially consistent, *Self, r*(23) = .28, *p* = .182; *Friend, r*(23) = .20, *p* = .347; *Stranger, r*(23) = .58, *p* = .002.

#### Prioritization biases

**Figure 1.**
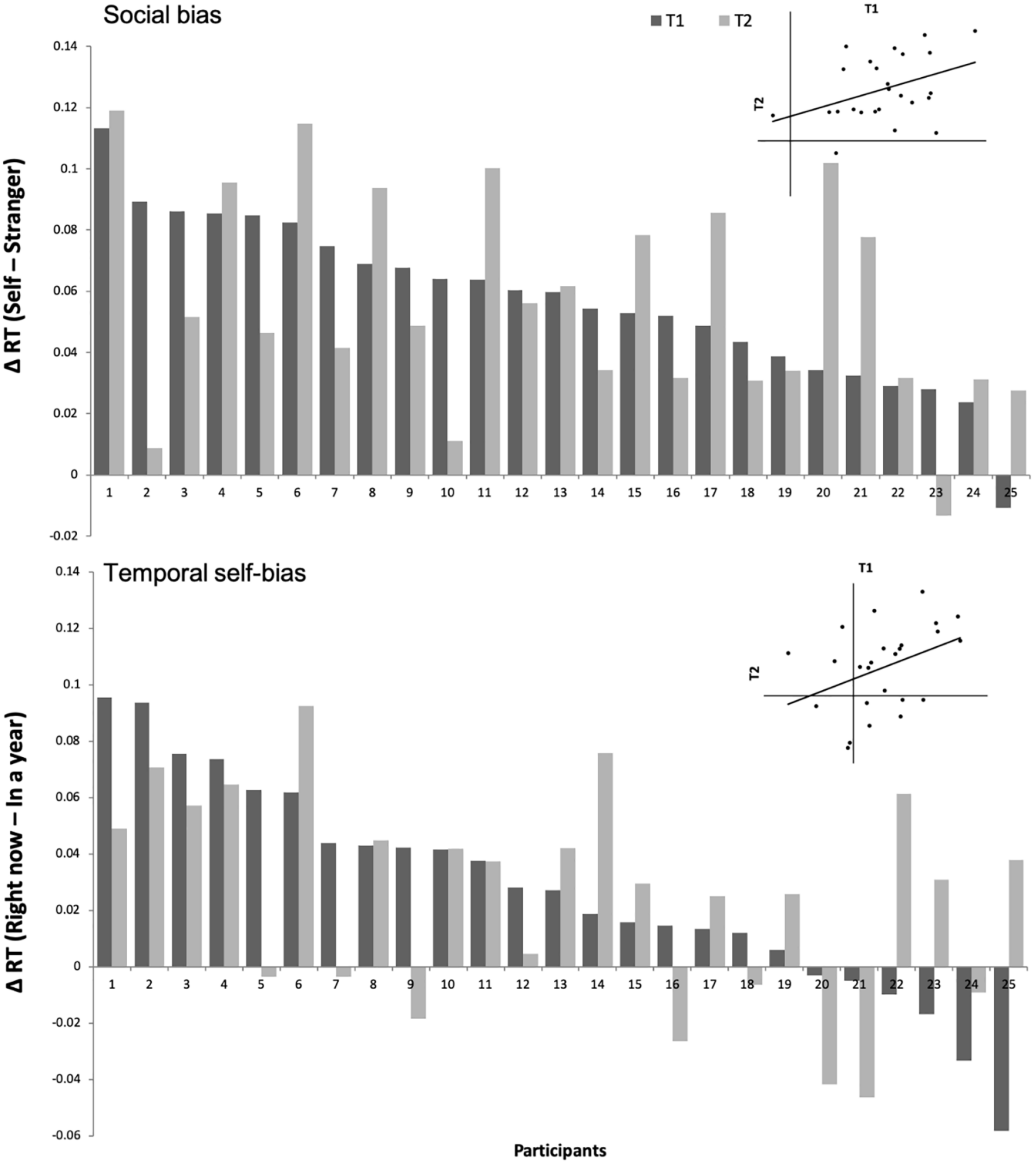
Individual social and temporal self-biases in T1 and T2 measured in Experiments 1a and 1b.

Social and temporal self-biases were calculated in the same way with normalized RTs as per Experiment 1a. To test for the stability of the individual temporal self-bias over time, we performed a Bayesian one sample *t*-test with a variable calculated by subtracting the temporal self-biases (*Right now*—*In a year*) at T1 from T2. The analysis revealed that our null hypothesis (no change) was supported 4.64 (BF_01_; BF_10_ = 0.22) times more than the alternative hypothesis (change over time) at the 95% credible interval of [−0.015, 0.019], *se* = 0.008, indicating that participants’ temporal self-biases were unchanged. For the individual social self-bias (*Self*—*Stranger*), a Bayesian one sample *t*-test revealed that our null hypothesis (no change) was also supported 4.74 (BF_01_; BF_10_ = 0.21) times more than the alternative hypothesis (change over time) at the 95% credible interval of [−0.015, 0.014], *se* = 0.007, indicating that participants’ social self-biases were also unchanged. Significant correlations were found between T1 and T2 on the temporal self-biases, *Tomorrow*—*Right now: r*(23) = .50, *p* = .011; *In a year*—*Right now: r*(23) = .39, *p* = .054; *In a year*—*Tomorrow: r*(23) = .41, *p* = .043, but only partially significant correlations were found on the social biases, *friend*—*Self: r*(23) = –.16, *p* = .461; *Stranger*—*Self: r*(23) = .39, *p* = .055; *Stranger*—*Friend: r*(23) = –.10, *p* = .640. Inter-temporal changes on the social and temporal self-biases for the extreme immediate and expanded self-associated shapes per individual are depicted in Figure 1. Our results indicated that the social and temporal self-biases remained largely consistent over 4 months.

Based on our findings, implicit prioritization biases toward immediate self-relevant information appeared to be a common trend across individuals that can last over time and the magnitudes of such biases were generally consistent. Overall, our findings imply that the information prioritization effects might be enduring characteristics for generally differentiating immediate from expanded self-information in the temporal and social domains. And the prioritization effects seemed to be more a trait-like rather than a state-like measure.

Next, we explored a spatial domain and compared individual differences of the spatial self-bias with the temporal self-bias where we found the most robust stability across individuals over time.

## Experiment 2. Prioritizing immediate spatial and temporal selves

In Experiment 2, we investigated whether people would prioritize immediate spatial self-information (e.g., me right here) over expanded spatial self-information (e.g., me far away) and whether such a prioritization tendency would be associated with the individual temporal self-bias.

### Method

#### Participants

Thirty-eight participants (4 males, *M*_age_ = 20.33 years, *SD*_age_ = 2.95 years) were recruited for an exchange of course credits at the University of Vienna. Based on our effect size observed in Experiment 1a, a minimum sample size to detect the prioritization effect with 80% power at the alpha level of 0.05 in temporal task was 20.

#### Stimuli and task

Six geometric shapes were randomly assigned to two sets of labels: spatial self-labels: me *Here, There, Far away*; temporal self-labels: me *Right now, Tomorrow, In a year*. The tasks were employed in the same way as in Experiment 1a.

#### Procedure

Participants were instructed to make associations between shapes and spatial or temporal self-labels. Example associations participants read for spatial self-labels are “Cross is myself right here,” “Hexagon is myself over there,” and “Horizontal ellipse is myself far away.” The order of presenting associations was counterbalanced across participants for both spatial and temporal tasks. The rest of the procedure followed as per Experiment 1a.

### Results and discussion

Identical analyses to Experiment 1a were performed.

#### Processing speed (RTs)

Two separate 2 (matching vs. nonmatching) × 3 (shape category) ANOVAs on RTs revealed significant interactions between matching condition and shape category on RTs in the spatial, *F*(2, 74) = 26.63, *p* < .001, $\eta_{p}^{2}=.46$ , and in the temporal domains, *F*(2, 74) = 11.26, *p* < .001, $\eta_{p}^{2}=.23$ (see Table 3 for the means). Significant effects of shape category emerged for correct matching trials in the spatial, *F*(1.56, 57.79) = 45.17 (Greenhouse–Geisser corrected), *p* < .001, $\eta_{p}^{2}=.55$ , and the temporal domains, *F*(2, 74) = 5.39, *p* = .007, $\eta_{p}^{2}=.13$ , indicating that participants prioritized immediate over expanded self-associated shapes in both domains. Multiple comparisons revealed largely significant differences between all spatial and temporal shape categories (see Table 3).

**Table 3.** Mean reaction times and accuracies as a function of matching condition (matching vs. non-matching) and shape category in Experiment 2 (*N* = 38).

Task	Conditions	Shape category	Mean RT (ms)	Accuracy
Spatial self-labels	Matching	Here	626 (78)_a b_	0.89 (0.07)
		There	690 (94)_a c_	0.80 (0.11)
		Far away	706 (103)_b c_	0.79 (0.15)
	Non-matching	Here	737 (79)	0.80 (0.16)
		There	750 (82)	0.78 (0.16)
		Far away	744 (84)	0.81 (0.15)
Temporal self-labels	Matching	Right now	666 (83)_d e_	0.86 (0.13)
		Tomorrow	677 (83)_d f_	0.83 (0.12)
		In 1 year	695 (67)_e f_	0.79 (0.13)
	Non-matching	Right now	735 (88)	0.79 (0.15)
		Tomorrow	755 (75)	0.80 (0.11)
		In 1 year	732 (80)	0.83 (0.12)

RT = reaction time; Accuracy = proportion correct. Standard deviations appear within parentheses.

The following *p* values assigned to each subscript represent pairwise comparisons between conditions with the shared subscript. ^a,b^*p* < .001. ^c^*p* = .053. ^d^*p* = .075. ^e^*p* = .005, ^f^*p* = .261. All ps were FDR-corrected.

#### Performance (*d′*)

Significant effects of shape category emerged in the spatial, *F*(2, 74) = 12.42, *p* < .001, $\eta_{p}^{2}=.25$ , but not in the temporal domain, *F*(2, 74) = 2.17, *p* = .122, $\eta_{p}^{2}=.06$ (spatial shape category: *Here: M* = 2.28, *SD* = .88; *There: M* = 1.80, *SD* = .75; *Far away: M* = 1.93, *SD* = .94); temporal shape category: *Right now: M* = 2.15, *SD* = 1.00; *Tomorrow: M* = 1.98, *SD* = .79; and *In a year: M* = 1.95, *SD* = .90). Significant differences were found between *Here*—*There* and *Here*—*Far away* comparisons (FDR-corrected *p*s ⩽ .001, 002; *There*—*Far away, p* = .206), and the differences between the *Right now*—*Tomorrow* and *Right now*—*In a year* comparisons were not significant (FDR-corrected *p*s = .10; *Tomorrow*—*In a year, p* = .749). A further 2 (domain) by 3 (shape category) Bayesian repeated measures ANOVA revealed that our data moderately supported the null hypothesis (H0) against the effect of domain (BF_10_ = 0.151) and also against the interaction effect between domain and shape category (BF_10_ = 0.317), indicating that our participants showed similar sensitivity differences among temporal self- compared to spatial self-labels.

#### Prioritization biases

**Figure 2.**
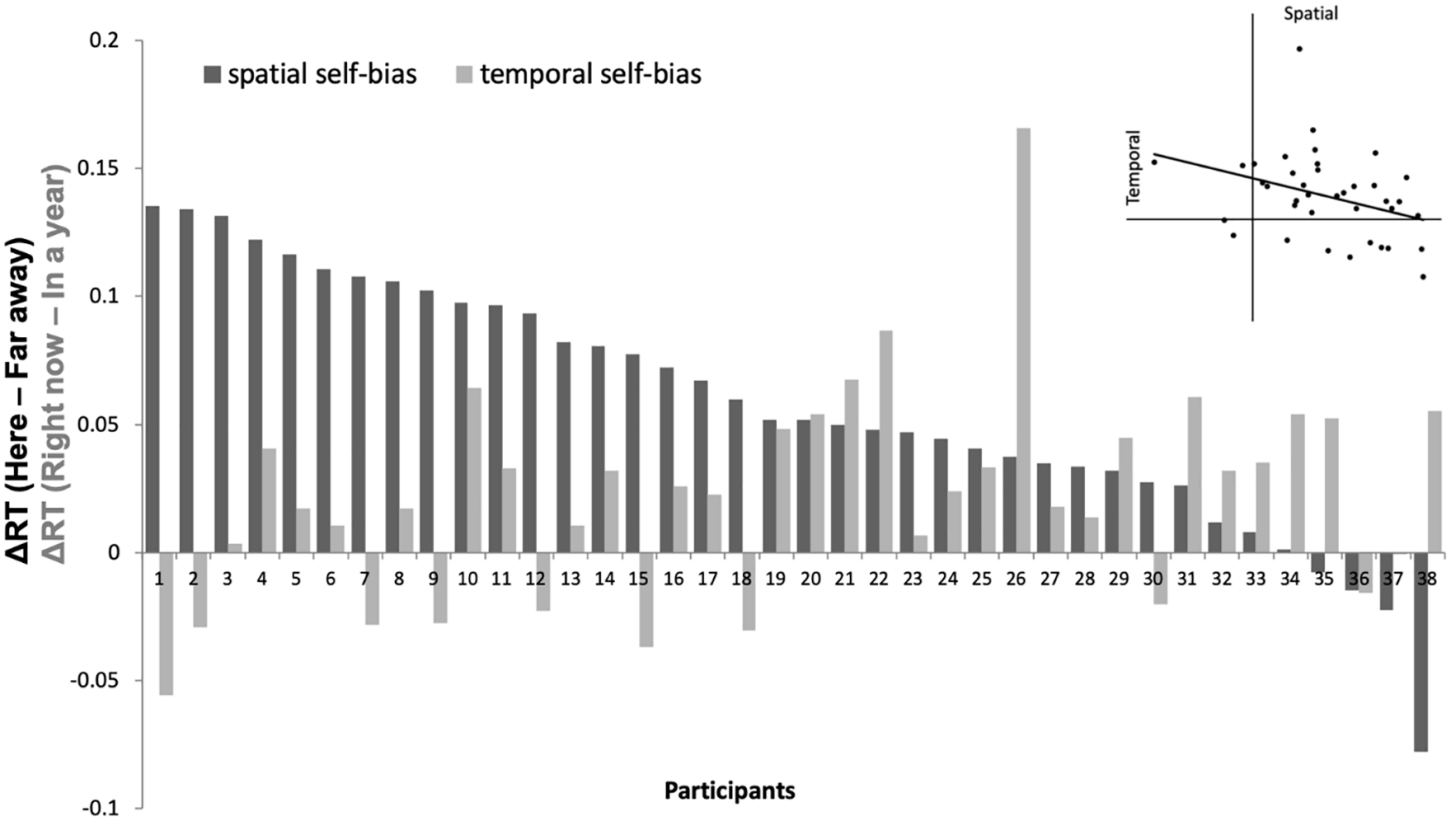
Individual spatial and temporal self-biases measured in Experiment 2.

Prioritization biases were calculated by subtracting the mean RTs of immediate self-associated shapes from RTs of expanded self-associated shapes and dividing it by the sum of two mean RTs for forming spatial (*Far away*—*Here*) and temporal (*In a year*—*Right now*) self-biases. Correlational results showed that overall, the temporal self-bias was negatively correlated with the spatial self-bias, *r*(36) = –.35, *p* = .03, also at the bias levels between *Tomorrow*—*Right now* and *There*—*Here, r*(36) = –.40, *p* = .01 (see supplementary material for full correlational results between all bias levels), indicating that the more temporal self-bias participants had, the less spatial self-bias participants showed and vice versa. Individual spatial and temporal biases per participant are depicted in Figure 2.

In Experiment 2, we showed that the prioritization effect occurred in the spatial domain in a way that people systematically prioritized immediate (i.e., “me here”) over expanded (i.e., “me far away”) self-information. More interestingly, the magnitude of the spatial self-bias was negatively associated with the magnitude of the temporal self-bias, quite consistently at each bias level. This finding suggests that temporal and spatial self-representation might not be completely separate constructs for processing self-relevant information. The negative association might indicate that the temporal and spatial self-concepts are represented on a considerable overlap whereby one type of bias could be more dominant than another within an individual. For instance, people who show a stronger temporal self-bias might show less differential processing for spatially expanded selves (i.e., me here, me far away) and those who show a stronger spatial self-bias might show less differential processing for temporally expanded selves. More research is in need to verify this representational overlap between the temporal and spatial self-concepts. Finally, unlike in Experiment 1a, the significant prioritization effect for “*Right now*” compared to “*Tomorrow*” shape category was absent indicating that the bias effects between these two shape categories were inconsistent, although the overall prioritization effects were significant.

Next, we explored a probability domain of self-relevant information to test whether the prioritization effect occurs in the probability domain of self-representation. We also tested whether participants’ individual probable self-bias is associated with their temporal self-bias.

## Experiment 3. Prioritizing immediately probable and temporal selves

In Experiment 3, we investigated whether people prioritize immediately probable self-information (e.g., “me for sure”) over expanded probable self-information (e.g., “me maybe”) and whether such a prioritization bias is associated with participants’ individual temporal self-bias.

### Participants

Forty participants (9 males, *M*_age_ = 21.45 years, *SD*_age_ = 2.99 years) were recruited for an exchange of course credits at the University of Vienna. Based on the averaged effect size observed in Experiments 1a and 2, a minimum sample size to detect the prioritization effect with 80% power at the alpha level of .05 in the temporal task was 25.

### Stimuli and task

Six geometric shapes were randomly assigned to two sets of labels: probable self-labels: me *Surely, Likely, Maybe*; temporal self-labels: me *Right now, Tomorrow, In a year*. The tasks were employed in the same way as in Experiment 1a.

### Procedure

Participants were instructed to make associations between shapes and probable or temporal self-labels. Example associations participants read for probable self-labels are “Cross is surely myself,” “Hexagon is likely myself,” and “Horizontal ellipse is maybe myself.” The order of presenting associations was counterbalanced across participants for both probability and temporal tasks. The rest of the procedure followed as per Experiment 1a.

### Results and discussion

#### Processing speed (RT)

Two separate 2 (matching vs. nonmatching) × 3 (shape category) ANOVAs on RTs revealed a non-significant interaction between matching condition and shape category in the probability domain, *F*(2, 78) = 2.41, *p* = .097, $\eta_{p}^{2}=.06$ , but a significant interaction in the temporal domain, *F*(2, 78) = 8.13, *p* = .001, $\eta_{p}^{2}=.17$ (see Table 4 for the means). The effect of shape category was marginally significant for correct matching trials in the probability domain, *F*(2, 78) = 2.52, *p* = .087, $\eta_{p}^{2}=.06$ , but a significant effect was found in the temporal domain, *F*(2, 78) = 9.92, *p* < .001, $\eta_{p}^{2}=.20$ , indicating that participants prioritized immediate over expanded associated shapes in the probability and temporal domains. Multiple comparisons revealed significant differences between only one probability shape category pair (*Surely*—*Likely*) but between all temporal shape category pairs (Table 4). Overall, participants prioritized immediate over expanded self-information partially in the probability domain and fully in the temporal domain.

**Table 4.** Mean reaction times and accuracies as a function of matching condition (matching vs. non-matching) and shape category in Experiment 3 (*N* = 40).

Task	Conditions	Shape category	Mean RT (ms)	Accuracy
Probable self-labels	Matching	Surely	631 (95)_a b_	0.81 (0.13)
		Likely	657 (114)_a c_	0.75 (0.16)
		Maybe	645 (117)_b c_	0.75 (0.18)
	Non-matching	Surely	713 (120)	0.71 (0.18)
		Likely	710 (124)	0.70 (0.17)
		Maybe	712 (130)	0.81 (0.16)
Temporal self-labels	Matching	Right now	634 (93)_d e_	0.84 (0.15)
		Tomorrow	660 (97)_d f_	0.79 (0.14)
		In 1 year	678 (98)_e f_	0.73 (0.15)
	Non-matching	Right now	720 (126)	0.68 (0.16)
		Tomorrow	731 (140)	0.74 (0.15)
		In 1 year	719 (118)	0.75 (0.17)

RT = reaction time; Accuracy = proportion correct. Standard deviations appear within parentheses.

The following *p* values assigned to each subscript represent pairwise comparisons between conditions with the shared subscript. ^a^*p* = .039. ^b,c^*p* = .318. ^d^*p* = .03. ^e^*p* = < .001. ^f^*p* = .048. All ps were FDR-corrected.

#### Performance (*d*′)

Two separate ANOVAs revealed a significant main effects of shape category on performance sensitivity in the probability domain, *F*(2, 78) = 3.69, *p* = .029, $\eta_{p}^{2}=.09$ , and a marginal effect in the temporal domain, *F*(2, 78) = 2.73, *p* = .072, $\eta_{p}^{2}=.07$ . FDR corrected comparisons revealed a significant *Surely*—*Likely* comparison (*p* = .028, *Surely*—*Maybe: p* = .085, *Likely*—*Maybe: p* = .848) and a significant *Right now*—*In a year* comparison (*p* = .023, *Right now*—*Tomorrow: p* = .588, *Tomorrow*—*In a year: p* = .142), indicating that the processing advantage of immediate over expanded self-information was largely supported but somewhat partially in performance sensitivity. A further 2 (domain) by 3 (shape category) Bayesian repeated measures ANOVA revealed that our data slightly supported the null hypothesis against the effect of domain (BF_10_ = 0.709) and moderately supported the null hypothesis against the interaction effect between domain and shape category (BF_10_ = 0.180), indicating that our participants showed similar sensitivity differences among temporal self- compared to probable self-labels.

#### Prioritization biases

Prioritization biases were calculated by subtracting the mean RTs of the immediate self-associated shapes from the RTs of the expanded self-associated shapes for the probable (*Likely*—*Surely*) and temporal (*In a year*—*Right now*) self-biases. Correlational results showed no significant association between these two self-biases, *r*(38) = .11, *p* = .51 (see supplementary material for full correlational results).

In Experiment 3, we showed that participants systematically prioritized immediate (i.e., “Surely me”) over expanded (“Likely me”) self-information in the probability domain but such a bias did not correlate with the temporal self-bias. Once again, our findings confirmed that people might have a general tendency to prioritize self-relevant information that is more immediate than expanded from the experiential self across multiple domains of self-representation. However, the magnitudes of such prioritization biases varied across domains at the individual level.

## General discussion

As hypothesized, our results consistently showed that participants prioritized immediate over expanded self-information in temporal, social, spatial, and probability domains. The magnitudes of prioritizing immediate over expanded self-information in the temporal and social domains lasted over time (4 months; Experiment 1b), indicating stable characteristics for processing self-relevant information that expands to future and to other people. Nevertheless, the magnitudes of the prioritization effects did not consistently correlate with each other between the temporal and social, nor between the temporal and probability domains, implying possibly separate underlying mechanisms. A negative correlation was found between the temporal and spatial domains, suggesting that temporal and spatial self-representations might not be completely separate constructs.

**Figure 3.**
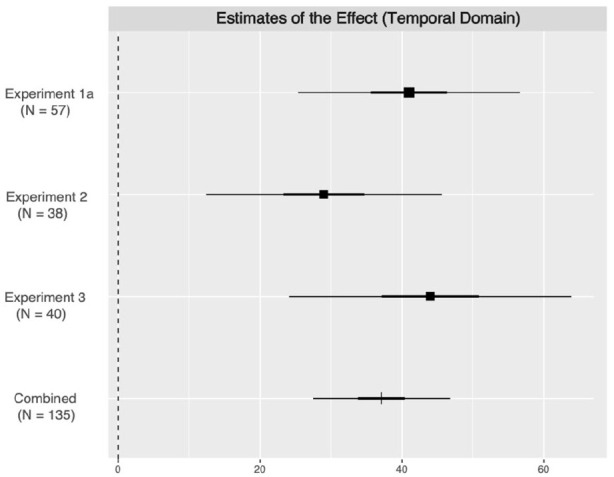
Estimates of immediate over expanded temporal self-bias effects (meta-analysis). Estimates of effects on the temporal self-bias in Experiments 1a, 2, and 3. Two ranges of bar at each point of estimates indicate 50% and 95% confidence intervals.

To further examine the robustness of the prioritization effect for the temporal domain observed in our experiments, we conducted a meta-analysis for testing the effects of me *Right now* and me *In a year* associated shapes on mean RTs of correct matching trials across Experiments 1a, 2, and 3, using the online tool provided by McShane and Böckenholt (2017). The results revealed a significant prioritization effect for *Right now* over *In a year* self-associated shapes, estimates of the effect at 37.37, 95% CI = [27.66, 47.08], *SE* = 4.95, *z* = 7.56, *p* < .001. The meta-analysis indicated that the prioritization effect for the temporal-self-bias observed in the present research is highly robust (Figure 3).

Finally, as each of our experimental procedure included the identical shape–label matching task for the temporal self-labels, we collated our data across three studies (Experiments 1a, 2, and 3; *N* = 135) to assess internal consistency of measuring the temporal self-bias across three blocks within a task. Recall that these three blocks were always randomly interleaved with three other blocks testing for another domain. We first normalized the RT differences for each pair of three temporal shape categories and tested for reliability across three blocks. The reliability test showed a considerably high internal consistency across three blocks measuring the temporal self-bias for *Right now* associated shapes contrasted to *In a year* associated shapes (α = .74). The temporal self-bias for *Right now* associated shapes contrasted to *Tomorrow* showed a lower but still reliable consistency (α = .66; *Tomorrow*—*In a year*: α = .52). All in all, our analyses indicated that the shape–label matching task for measuring the temporal self-prioritization tendency is fairly reliable. Given the high stability of the temporal self-bias over time (*r* = .50) observed in Experiment 1b, we recommend future researchers to utilize the current paradigm for gauging the individual temporal self-bias in information prioritization.

Overall, our findings provide strong evidence that the self-bias at the early information processing stage does not only apply to the social domain but also to the temporal and spatial domains, and also to a limited sense, to the probability domain. Accumulative research has shown that self-relevant information is generally processed faster, more accurately (Keyes & Dlugokencka, 2014; Sui & Han, 2007) and remembered better (e.g., Conway & Pleydell-Pearce, 2000; Symons & Johnson, 1997) than other-relevant information. However, whether a similar bias would occur within self-relevant information in intrapersonal domains was unclear. Our data clearly showed that people tend to prioritize self-information that is closer to the currently experienced self in time, space, and probability, indicating a systematic processing mechanism for differentiating immediate and expanded self-information at an early information processing stage. The current findings represent a novel discovery of how the self as subject (experiencing self) and the self as object (content-based self) might be differentiated at the representational level beyond the social domain. Our results are in line with the basis model of self-specificity (Northoff, 2016) in a way that the self as subject might be the fundamental grounding of the self-concept (thus most salient) rather than a higher order meta-representational function. Similarly, the immediate self-prioritization effect also supports the spatiotemporal view of the self (Northoff, 2016) claiming that the self arises not only as a social entity but also as an integrative construct in temporal and spatial features of processes.

In a more elaborated social domain, recent evidence showing biases toward different self-identities (e.g., morally good vs. bad self; Golubickis et al., 2019; Hu et al., 2020) and self-bias overlapping with the process of in-group-bias (Enock et al., 2018) also contribute to a better understanding of the self-bias within a wider range of self-representation. Further research is encouraged to identify at which process such prioritization might occur (e.g., encoding, retrieval or earlier perceptual stage) and whether differential association-learning effects might occur across domains. Whether such prioritization effects persist when information processing is hindered (e.g., with a temporal delay between presenting a shape and a label; Kolvoort et al., 2020) could answer to what extent prioritization effects for immediate self-relevant information occur.

So far, empirical evidence has shown analogical consequences for processing temporally and socially expanded selves at a higher level of information processing (i.e., judgments and decision-making): People tend to discount future monetary rewards for themselves as much as they would discount the value of money to forgo on behalf of a socially distant person (Rachlin & Jones, 2008); Future decisions for oneself often resemble those we make for other people (Pronin et al., 2008; Pronin & Ross, 2006); People construe temporally, socially, spatially, and hypothetically distant information in a more abstract and general way than proximate information (Trope & Liberman, 2010); Mentalizing with others, prospecting future, and remembering past events share a cognitive process such as episodic simulation to understand other people and plan future actions (Schacter et al., 2007). Evidently, various lines of previous research have constantly pointed to a uniquely shared mechanism for processing the currently experiencing self as opposed to expanded selves across multiple domains. Yet, to date, through what mechanism the shared process rises at an early information processing stage was unknown. By utilizing a behavioral paradigm to measure a specific processing advantage, we have identified implicit prioritization biases and demonstrated that self-relevant information is systematically prioritized as a function of closeness to the current self.

Interestingly, our results also showed that the magnitudes of prioritizing immediate over expanded self-information in temporal, social, and probability domains did not consistently correlate with each other suggesting separate underlying mechanisms for processing self-information that stretches to temporal, social, and probability domains. This pattern might be revealing the structural differences that distinguish temporal selves from social or probable selves in self-representation. However, further empirical evidence is in need to verify precise representational overlaps and boundary conditions between currently tested domains. In sum, our research suggests that, although immediate and expanded self-information might be processed in a similar fashion, the magnitudes of the representational overlaps for immediate and expanded self-information across temporal, social, and probability domains might differ within individuals.

The present research is in line with a body of research claiming that the self-identity operates as a motivating and goal-directing factor (e.g., Berkman, Livingstone et al., 2017). We argue that the function of the systematic prioritization effects observed in the present study is to process information that is most salient in an efficient way. In fact, depending on the individual needs and goals, the level of significance to self-relevant information might vary (e.g., Kim & Johnson, 2012, 2014). Thus, although self-relevant information in general might carry a certain level of salience what is most salient at a given moment might differ across individuals and across domains. Consequently, people who tend to prioritize the self *Right now* might identify themselves more with who they are at the present moment, whereas people who tend to prioritize the self *In a year* from now might identify themselves more with who they want to be in a year. Based on this logic, future research might unravel whether the representational overlap between the current self and the future self can modulate the level of the temporal self-bias. Another interesting line of research would be to investigate decision contexts that might be able to temporarily switch individual’s bias direction at a given moment (prioritizing now vs. prioritizing future). Finally, whether domain-specific prioritizations occur depending on how much people identify themselves in one particular over other domains and whether the strength of such domain-specific biases relates to the level of self-identification in the same domain are important future research questions to be answered.

Finally, one of the limitations of the present research is that although the salience account might be the most likely explanation of the currently observed prioritization effects, salience was not directly measured. However, we consider processing speed and accuracy as an index of salience in information processing based on previous research using the same paradigm (e.g., Sui et al., 2012). Another limitation is that, although our results showed that the prioritization effects were mostly uncorrelated, our results do not speak to the interaction of the main effects between domains because the baselines for each domain are not directly comparable (i.e., stranger, me in a year, me far away, me maybe). Future research can test for more direct interaction effects, for instance, whether the information prioritization would show an additive trend when two or three different domains are combined. Overall, we encourage future researchers to verify further novel processing advantages for immediate over expanded self-information using different behavioral paradigms and test for representational overlaps across multiple domains to constitute an integrative structural framework of self-representation in the field.

## Supplemental Material

sj-docx-1-qjp-10.1177_17470218211004208 – Supplemental material for Immediate self-information is prioritized over expanded self-information across temporal, social, spatial, and probability domains Supplemental material, sj-docx-1-qjp-10.1177_17470218211004208 for Immediate self-information is prioritized over expanded self-information across temporal, social, spatial, and probability domains by Hyunji Kim and Arnd Florack in Quarterly Journal of Experimental Psychology
