# Supplementary material for: Immediate self-information is prioritized over expanded self-information across temporal, social, spatial, and probability domains
Source: Q J Exp Psychol (Hove). 2021 Apr 2;74(9):1615–30. doi: 10.1177/17470218211004208 (PMC8358571; doi:10.1177/17470218211004208)
Supplement: sj-docx-1-qjp-10.1177_17470218211004208 – Supplemental material for Immediate self-information is prioritized over expanded self-information across temporal, social, spatial, and probability domains [file sj-docx-1-qjp-10.1177_17470218211004208.docx]

Supplementary Material for:

**Immediate Self-information is Prioritized over Expanded Self-information**

**across Temporal, Social, Spatial, and Probability Domains**

Hyunji Kim

Arnd Florack

**S1 Table. German translated labels used in Experiments 1a to 3.**

|  | English | German |
| --- | --- | --- |
| Temporal self-labels | Right now, Tomorrow,  In 1 year | Jetzt gerade, Morgen, In einem Jahr |
| Social labels | Self, Friend, Stranger | Selbst, Freund, Fremder |
| Spatial self-labels | Here, There, Far away | Hier, Nebenan, Weit weg |
| Counterfactual self-labels | Surely, Likely, Maybe | Ganz sicher, Wahrscheinlich, Vielleicht |

**S2 Table. Inter-correlations between social and temporal self-biases in Experiment 1a (N = 57).**

|  | 1. | 2. | 3. | 4. | 5. | 6. |
| --- | --- | --- | --- | --- | --- | --- |
| 1. Self-Friend | -- |  |  |  |  |  |
| 2. Self-Stranger | .57** | -- |  |  |  |  |
| 3. Friend-Stranger | -.31* | .61** | -- |  |  |  |
| 4. Now-Tomorrow | -.02 | -.05 | .12 | -- |  |  |
| 5. Now-One year | -.07 | -.05 | .02 | .66** | -- |  |
| 6. Tomorrow-One year | -.07 | -.15 | -.11 | -.23 | .58** | -- |

* *p* < .05 ** *p* < .001

**Experiment 1b**

**Performance (*d´*).** Significant effects of shape category emerged in the social, *F*(1.59, 38.08) = 37.03, *p* < .001, *η*_p_^2^ = .61, and in the temporal domain, *F*(1.55, 37.14) = 6.34, *p* = .008, *η*_p_^2^ = .21 (Greenhouse-Geisser corrected *df*s; see S3 for means for T2). Significant differences were found between all social pairs (FDR-corrected *p*s ≤ .003) and two temporal pairs *Right now* – *Tomorrow* (*p = .*023) and *Tomorrow* – *In a year* (*p = .*023) but not in the *Right now* – *In a year* pair (*p = .*171). A further 2 (dimension) by 3 (shape category) ANOVA revealed that an interaction between the dimension and shape category was not significant, *F*(2, 48) = 21.05, *p* < .001, *η*_p_^2^ = .47, implying a stronger bias for the socially immediate compared to temporally immediate shape category.

**S3 Table. *d´* results in T1 and T2 and T-Test results in Experiment 1b.**

| Task |  | Shape category | T1: *M* (*SD*) | T2: *M* (*SD*) | Paired samples t-tests between T1 and T2 |
| --- | --- | --- | --- | --- | --- |
| Social labels |  | *Myself* | 2.46 (0.92) | 2.76 (0.95) | *t*(24) = 1.33, *p* = .195 |
|  |  | *Friend* | 1.86 (1.01) | 2.02 (0.79) | *t*(24) = 0.67, *p* = .51 |
|  |  | *Stranger* | 1.43 (0.74) | 1.72 (0.73) | *t*(24) = 2.14, *p* = . 043 |
| Temporal self-labels |  | *Right now* | 2.02 (0.69) | 2.15 (0.64) | *t*(24) = 1.30, *p* = .207 |
|  |  | *Tomorrow* | 1.97 (0.63) | 2.43 (0.68) | *t*(24) = 3.34, *p* = .003 |
|  |  | *In a year* | 1.74 (0.71) | 2.01 (0.70) | *t*(24) = 1.74, *p* = .095 |

**S4 Table. Inter-correlations between temporal and spatial self-biases in Experiment 2 (N = 38).**

|  | 1. | 2. | 3. | 4. | 5. | 6. |
| --- | --- | --- | --- | --- | --- | --- |
| 1. Now-Tomorrow | -- |  |  |  |  |  |
| 2. Now-One year | .54** | -- |  |  |  |  |
| 3. Tomorrow-One year | -.50* | .47** | -- |  |  |  |
| 4. Here-There | -.40* | -.50** | -.10 | -- |  |  |
| 5. Here-Far away | -.10 | -.35* | -.26 | .66** | -- |  |
| 6. There-far away | .25 | .01 | -.25 | -.10 | .69** | -- |

* *p* < .05 ** *p* < .01

**S5 Table. Inter-correlations between temporal and probable self-biases in Experiment 3 (N = 40).**

|  | 1. | 2. | 3. | 4. | 5. | 6. |
| --- | --- | --- | --- | --- | --- | --- |
| 1. Now-Tomorrow | -- |  |  |  |  |  |
| 2. Now-One year | .60** | -- |  |  |  |  |
| 3. Tomorrow-One year | -.50** | .43** | -- |  |  |  |
| 4. Surely-Likely | .10 | .11 | .004 | -- |  |  |
| 5. Surely-Maybe | -.11 | -.35* | -.27 | .63** | -- |  |
| 6. Likely-Maybe | -.24 | -.55** | -.34* | -.20 | .63** | -- |

* *p* < .05 ** *p* < .01
